# Acupuncture after surgery of soft tissue tumors of the extremities: postoperative pain and feasibility: a prospective trial

**DOI:** 10.1007/s00423-026-04365-z

**Published:** 2026-11-10

**Authors:** Erfan Ghanad, Hannah Fischer, Christel Weiß, Martin Dusch, Franka Menge, Helene Weigl, Madelaine Hettler, Melissa Harbrücker, Niki Taebi, Christoph Reissfelder, Cui Yang, Jens Jakob

**Affiliations:** 1 Department of Surgery, Medical Faculty Mannheim, University Medical Center Mannheim, Heidelberg University, Mannheim, Germany; 2 Department of Anesthesiology and Intensive Care, Pain Centre, Medical Faculty Mannheim, University Medical Center Mannheim, Heidelberg University, Mannheim, Germany; 3 Department of Medical Statistics and Biomathematics, Medical Faculty Mannheim, Heidelberg University, Mannheim, Germany; 4 Department of Surgery, Sarcoma Unit, Medical Faculty Mannheim, University Medical Center Mannheim, Heidelberg University, Theodor-Kutzer-Ufer 1-3, Mannheim, 68135 Germany; 5 DKFZ Hector Cancer Institute at the University Medical Center Mannheim, Mannheim, Germany

**Keywords:** Acupuncture, Postoperative pain, Extremity function, Soft tissue sarcoma, Lipoma, Pain management

## Abstract

**Purpose:**

Acute pain management following resection of soft tissue tumors (STTs) of the extremities is primarily based on pharmacological interventions. However, current guidelines emphasize the role of complementary non-pharmacological approaches, such as acupuncture. This prospective pilot study aimed to evaluate the feasibility and potential effects of acupuncture following STT surgery of the extremities. Furthermore, this study represents the first to systematically assess the postoperative pain trajectory in this patient population.

**Methods:**

In this prospective, single-arm, single-centre study, 25 patients undergoing surgical resection of STTs of the extremities received acupuncture on postoperative day (POD) 1. Pain intensity was recorded at predefined time points using the Numeric Rating Scale (NRS) and the Brief Pain Inventory (BPI).

**Results:**

Participants had a mean age of 60.7 years. Most tumors were malignant, located in the lower extremities, with a median size of 13.1 cm. Postoperative pain at rest was minimal, with a mean NRS score of 1.2 (± 1.2) on POD 1 and below 0.5 (± 0.8) from POD 2 onward. The pain severity index of the BPI declined significantly after POD 1, reaching its lowest level at POD 3. Acupuncture was administered on POD 1. Placement of needles at the “optimal position” was feasible in 8 of 25 patients. The median length of hospital stay was four days. Wound complications occurred in eleven patients, but none were attributable to acupuncture. No other serious adverse events were observed.

**Conclusion:**

Acupuncture was feasible, safe, and well accepted in this postoperative setting. Pain levels and opioid requirements remained low throughout the postoperative period. Further controlled studies are warranted to assess efficacy and to refine patient selection and acupuncture techniques in this setting.

**Trial registration:**

German Clinical Trials Register (DRKS, DRKS00032703) registeres on 18.09.2023.

## Introduction

Soft tissue tumors (STTs) are a heterogeneous group of neoplasms originating from mesenchymal tissue [1], and are classified into over 100 histological subtypes [2]. STTs can be benign or malignant, with benign forms occurring more frequentl [3]. Although benign STTs do not metastasize, they may cause significant morbidity through local growth and compression of adjacent structures and are typically managed by complete surgical resection [3]. In contrast, soft tissue sarcomas (STS) in adults are rare, with an incidence of approximately 5 per 100.000 inhabitants in Germany [4]. They primarily affect the extremities, grow destructively [3], and are treated with a combination of surgical resection and, if needed, radio- and/or chemotherapy to prevent recurrence and metastasis [5]. Regardless of histology, surgery remains the mainstay of treatment for most STTs.

Postoperative pain is a common phenomenon and one of the major concerns or fears of many patients prior to surgery [6, 7]. A prospective cohort study on burden and medical care of sarcoma in Germany (PROSa study) highlights, that functionality and quality of life are key concerns for sarcoma patients, with impairments particularly evident in the areas of pain (56%), fatigue (51%), emotional (63%), physical (60%) and role function (51%) [2].

While current acute pain management strategies rely predominantly on pharmacological treatments, analgesics may fail to provide adequate relief and can cause adverse effects. Non-opioid agents carry risks such as cardiotoxicity and nephrotoxicity, whereas opioids are associated with nausea, dizziness, and the potential for dependency [8, 9]. Consequently, current guideline recommendations underscore the increasing importance of non-pharmacological pain management strategies [10].

Acupuncture as an non-pharmacological modality is receiving increasing attention in the context of postoperative care [11–14] as well as in the treatment of cancer-related pain [15, 16]. German guidelines already recommend its integration into perioperative management for gastrointestinal tumors [17]. Acupuncture is applied in both acute and chronic pain management and is associated with minimal side effects [18–20]. A systematic review by Lu et al. reported that perioperative acupuncture may improve patient outcomes, including potential reductions in analgesic—particularly opioid—use, which in turn may accelerate the recovery of gastrointestinal function [21]. Preoperative acupuncture was also associated with lower postoperative pain scores and earlier mobilization following caesarean Sect [22]. Similarly, a meta-analysis by Sun et al. found that adjunctive acupuncture across various surgical settings was associated with reduced pain and opioid consumption. However, significant heterogeneity in study designs and outcome measures limits the comparability of existing evidence [23].

The present study aimed to evaluate the feasibility of acupuncture for managing postoperative pain following STT surgery of the extremities. To our knowledge, this was the first prospective study to investigate acupuncture in this specific patient population and to report postoperative outcomes in this context.

## Materials and methods

### Study design

This single-arm study was conducted from November 2023 till October 2024 at the Sarcoma Unit of the University Medical Centre Mannheim, Mannheim, Germany, which has been certified by the German Cancer Society for excellence in Sarcoma Care. Patients undergoing elective resection of STTs of the extremities were eligible.

### Recruitment and eligibility

Potential participants had to be scheduled for elective surgery for STTs of the extremities, either a benign tumor larger than 5 cm or a malignant one. Additional inclusion criteria were age ≥ 18 years and sufficient language proficiency for study participation.

Exclusion criteria included pregnancy, diabetic polyneuropathy, needle phobia, relevant psychiatric disorders, severe coagulation disorders (e.g., clinically significant haemophilia), inadequate communication skills, or previous amputation of the affected extremity.

### Standard of care

Tumor resections were performed according to current guidelines, with the surgical technique documented in each case [24].

The Sarcoma Unit follows an established postoperative care pathway for extremity STT resection, covering pain management, mobilisation, venous thromboembolism prophylaxis, and physiotherapy.

The standard analgesic regimen consisted of dipyrone (metamizole, 500 mg to 1 g four times daily, orally) or acetaminophen (paracetamol, 500 mg to 1 g four times daily, orally). Stronger analgesics, including opioids, were prescribed as needed. This protocol remained unchanged throughout the study, with acupuncture provided as an adjunct therapy.

### Intervention

An individualised acupuncture approach was used, tailored to the patient’s pain location. The intervention is reported in accordance with the Standards for Reporting Interventions in Controlled Trials of Acupuncture (STRICTA) criteria [25]:

1. Acupuncture rationale: Acupuncture was applied as typically performed in clinical practice, with points chosen based on Traditional Chinese Medicine (TCM) principles. Point selection was guided by TCM diagnostic criteria, including symptoms, palpation findings, tissue tenderness, pain associated with specific movements, and the surgical site.
2. Details of needling: Per session, approximately 4–12 sterile stainless steel needles (0.30 × 30 mm, TeWa, CE-certified) were inserted to a depth of 0.5–1.0 cm, depending on local tissue thickness. The De Qi sensation—described as soreness, heaviness, aching, or numbness[26] was elicited. Needles remained in situ for 20 minutes. Needle positions were documented photographically.
3. Treatment regimen: All participants received acupuncture on POD 1. A second session was performed on POD 3 if resting pain was ≥ 3/10 on the NRS.
4. Other components of treatment: No other TCM interventions were provided.
5. Practitioner background: The intervention was performed by a physician with several years of daily clinical acupuncture experience.
6. Control or comparator interventions: As this was an exploratory feasibility study, no control group was included.

### Study objective measurements

#### Primary objective measurements

To evaluate the primary objective, pain intensity was measured using the Numeric Rating Scale (NRS) and the Brief Pain Inventory (BPI) [27, 28]. The BPI, which assesses pain over the preceding 24 h, was completed once per visit, whereas the NRS score was additionally reassessed 20 min after acupuncture. Patients self-reported these values to minimise observer bias.

#### Secondary objectives measurements

The secondary objectives were evaluated as follows:

1. Pain assessment (NRS and BPI) was regularly performed within the postoperative setting.
2. The perioperative use of analgesics was recorded at each study visit.
3. Following surgical STT removal, a drain was often inserted for detection of postoperative bleeding or infection and for removal of wound secretion to support healing. The time point of drain removal was documented.
4. Upper extremity function was evaluated pre- and postoperatively using the Disabilities of the Arm, Shoulder and Hand (DASH) questionnaire, a validated and responsive tool that reliably detects changes in disability in musculoskeletal disorders [29, 30].
5. Lower extremity function was assessed using the Lower Extremity Functional Scale (LEFS), whose reliability, validity, and suitability for this patient group have been confirmed in a review by Mehta et al. [31].
6. Quality of life before surgery and during short-term follow-up was measured using the EORTC QLQ-C30 questionnaire, a widely used patient-reported outcome instrument in oncology research [32].
7. The length of hospital stay was recorded.
8. Compliance was monitored using a participant flow diagram adapted for single-arm studies according to the Consolidated Standards of Reporting Trials (CONSORT) guidelines, indicating reasons for non-participation and withdrawal.

### Sample size

As this was a feasibility study, a formal sample size calculation was not possible [33]. From an acupuncture perspective, it was challenging to define a standardised “one-size-fits-all” protocol. To include as many patients as possible with comparable tumor characteristics, the target sample size was set high. An initial recruitment goal of approximately 100 patients was determined to assess acupuncture effects across a heterogeneous STT cohort and to identify potential commonalities in acupuncture strategies across tumor sites and histologies.

### Statistical analysis

Statistical analyses were conducted using SAS software (version 9.4; SAS Institute, Cary, North Carolina, USA). Quantitative variables are reported as mean ± standard deviation for normally distributed data or as median and range for non-normal distributions. Pain scores were summarized using means, given their higher sensitivity to small differences compared with medians. Qualitative variables are presented as absolute and relative frequencies.

The primary and relevant secondary outcomes (quality of life and limb function questionnaires) were analysed using parametric or non-parametric tests, depending on data type and distribution. For normally distributed dependent samples, paired t-tests were applied; otherwise, the Wilcoxon signed-rank test was used. Baseline characteristics were summarised using descriptive statistics, and the same approach was applied to the remaining secondary outcomes. A p-value < 0.05 was considered statistically significant. Figures were created using GraphPad Prism version 10.3.

## Results

**Fig. 1:**
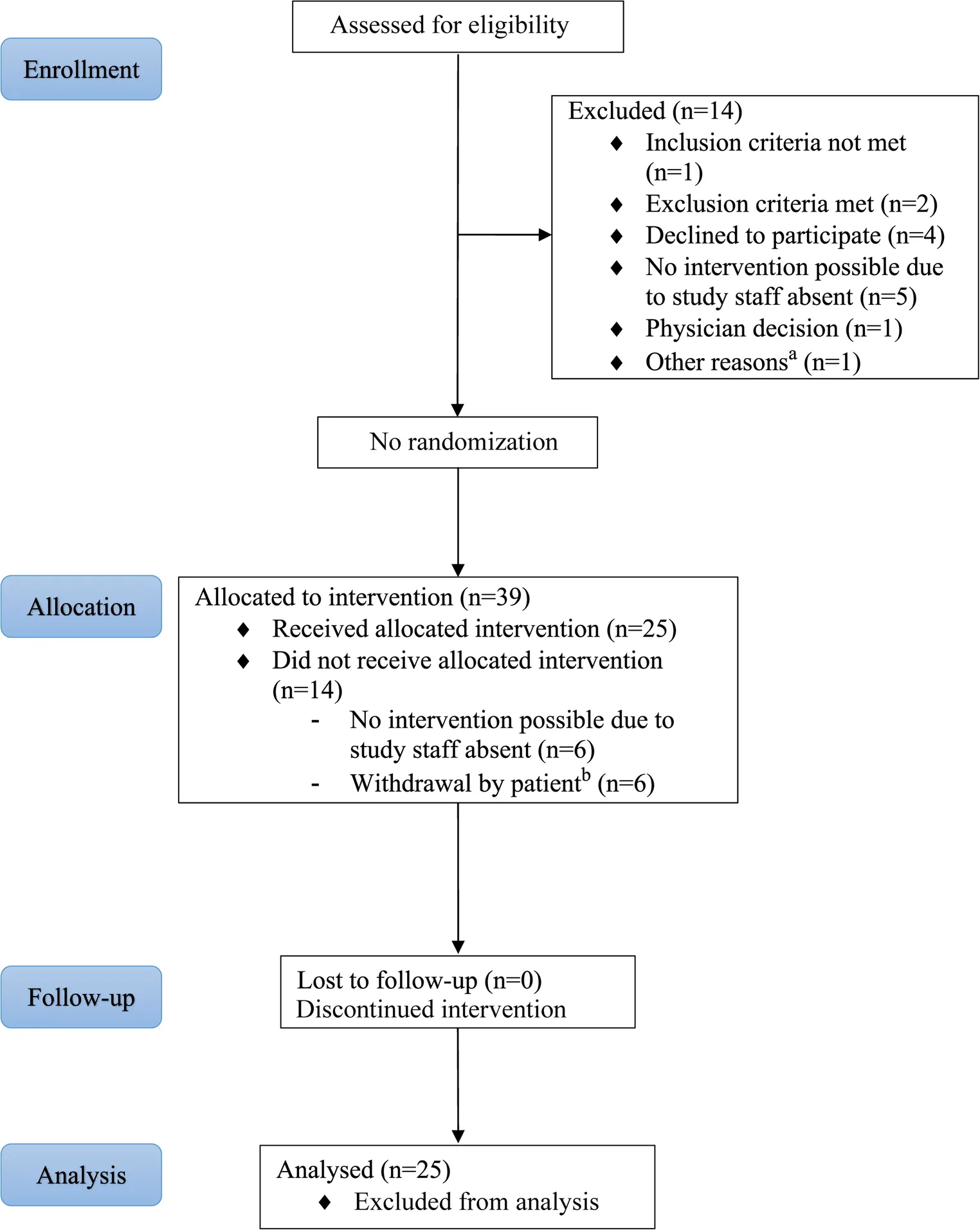
Flow-chart of the study according to CONSORT (Consolidated Standards of Reporting Trials). **a** One patient did not attend the informed consent discussion appointment for surgery. **b** Five patients decided against the intervention due to lack of postoperative pain, one patient perceived study participation as a burden. **c** Recruitment of patients was paused for interim analysis

Recruitment was conducted between November 2023 and September 2024. Of the 53 patients screened, 39 met the eligibility criteria and provided written informed consent (see Fig. 1). Ultimately, 25 patients received acupuncture treatment. The mean age at enrolment was 60.7 years, and the cohort showed a predominance of male participants. Baseline characteristics are presented in Table 1.

**Table 1:** Baseline characteristics

Age in years (mean ± SD )	60.7 (± 15.7)
Female	10 (40%)
Male	15 (60%)
BMI in kg/m^2^ (mean ± SD )	25.4 (± 3.7)
Tumor localization	
- Lower extremity	21 (84%)
- Upper extremity	4 (16%)
Core needle biopsy Preoperative incisional biopsy	19 (76%) 2 (8%)
Tumor size in cm (median, range)	13.1 (4.0–28.7)
Tumor subtypes /classification	
-Benign soft tissue tumors	3 (12%)
-Malignant soft tissue tumors	22 (88%)
Liposarcoma	9 (41%)
Undiff. pleomorph. sarcoma	3 (14%)
Other subtypes	10 (45%)
Preoperative chemotherapy	1 (4%)
Preoperative radiotherapy	6 (24%)
Regular alcohol consumption	9 (36%)
Smoking	2 (8%)
Median pain score at rest for all patients before surgery (median, range)	0 (0–6)
Numbers of patients with pain due to STT before surgery	8 (32%)
Regular pain medication before surgery	2 (8%)
Limitation of the extremity due to STTs before surgery	12 (48%)
STT related mobility restriction before surgery	2 (8%)
Experience with complementary medicine	
Homeopathy	9 (36%)
Phytomedicine	6 (24%)
Osteopathy	8 (32%)
Anthroposophical medicine	2 (8%)
Acupuncture	14 (56%)
Yoga / other meditations	3 (12%)
No experience	9 (36%)

*SD* Standard deviation, *BMI* Body Mass Index, *STT* Soft Tissue Tumor

**Fig. 2:**
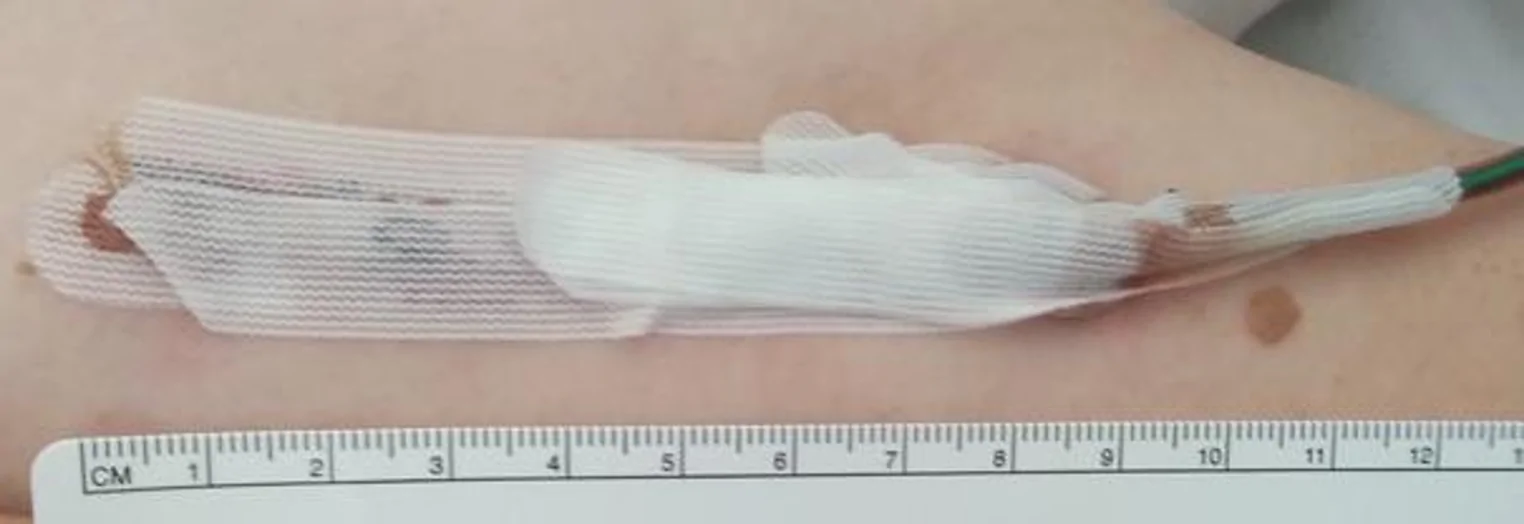
We developed this wound dressing method to allow the best possible access to manual body assessment and acupuncture points

The aim of the study was to assess the feasibility of acupuncture integration into perioperative care for patients undergoing surgical STT resection, with the objective of reducing postoperative pain and supporting recovery. Initially, the study aimed to recruit 100 participants; however, this target was revised downward for two main reasons. Postoperative pain was generally low, with mean NRS scores of 1.2 on POD 1 and < 1 on subsequent days. Second, full access to all relevant acupuncture points was often not feasible. Large wound dressings impeded the acupuncturist’s ability to assess the surgical area in some patients. In accordance with hygiene regulations, these dressings could not be removed on POD 1, limiting both palpation and accurate identification/stimulation of points located near the wound site. Following consultation with the hospital’s hygiene department, permission was granted to replace large dressings with sterile adhesive strips in select cases, thereby improving access to areas adjacent to the wound (see Fig. 2). Full access to relevant acupuncture points was possible in only 8 patients (32%). The most common barriers are summarized in Table 2, with the primary limitations being wound coverage by dressings, inability to lie in a prone position, and the presence of peripheral venous cannulas. Given these factors, an interim analysis was conducted after 25 patients were enrolled. Based on the findings, sufficient data was available to perform an exploratory analysis and to inform the design of future randomized follow-up studies.

**Table 2:** Impediment to effective acupuncture point accessibility

Optimal Acupuncture point selection possible	8 (32%)
Most important reasons for acupuncture point blockage:	f
-Large adhesive wound dressing	8
-Wound	7
-Abdominal positioning not possible	3
-VAC dressing applied	2
-Edematous tissue swelling	1
-Peripheral venous catheter	1
-Free flap from previous surgery	1
-Acupuncture too uncomfortable	1
-Pain due to palpation	1

*VAC* Vacuum-assisted closure

### Preoperative phase

Most STTs were malignant and predominantly located in the lower extremities. The median tumor size was 13.1 cm. Six patients had received radiotherapy, and one had received chemotherapy prior to surgery. Preoperatively, seven patients reported tumor site pain with an NRS score greater than 1, and two patients were receiving opioid analgesics. Nearly half of the cohort reported functional limitations of the affected extremity due to the STT before surgery, with two patients experiencing significant mobility restrictions.

### Surgery

Table 3 summarises the surgical characteristics. The mean procedure time was 68.4 min. A wide resection was performed in 72% of cases, with two cases requiring reconstruction using either a plastic or mesh graft. All but one patient had at least one surgical drain placed.

**Table 3:** Surgical procedures

Surgical procedure in minutes	68.4 (± 35.6)
Marginal excision	7 (28%)
Wide resection	18 (72%)
- Plus plastic reconstruction	1 (4%)
- Plus Mesh-graft	1 (4%)
Wound healing disorders	11 (44%)
- Wound dehiscence – secondary suturing, local anaesthesia (*n* = 1) and wound rinsed with sterile sodium chloride (*n* = 1) (CTCAE grade 2)	2 (8%)
- Aspiration of a seroma under local anesthesia (CTCAE grade 2)	1 (4%)
- Prolonged lymphocele formation :	
• Requiring extended evacuation of wound secretion by the intraoperatively placed drainages > 7 days (CTCAE grade 1) • Readmission for treatment of lymphocele (*n* = 1) or wound complication with VAC (*n* = 1) (CTCAE grade 3)	6 (24%) 2 (8%)
- Wound infection	0 (0%)
- Re-Operation	0 (0%)
- Other postoperative complications	0 (0%)
Lengths of hospital stay in days (median, range)	4 (1–18)
Numbers of drainages (median, range)	1 (0–3)
Drainage time in days (median, IQR, range)	4 (17, 1–33)
Rehospitalization	2 (8%)

*CTCAE* Common Criteria for Adverse Events, *VAC* Vacuum-assisted closure, *IQR* interquartile range

### Postoperative phase

During the postoperative period, pain levels remained low. The mean NRS pain score at rest was 1.2 on POD 1 and decreased to 0.2 by the day of discharge. Postoperative pain levels are summarized in Table 4. The majority of patients received opioids in the recovery room; however, use declined markedly thereafter, with only three patients requiring opioids on POD 1 and two patients on POD 3 (Table 5). On POD 3, nine patients reported no pain at rest (NRS = 0), whereas nineteen patients were pain-free at discharge and follow-up.

**Table 4:** Pain scores

	Preoperative	POD1	POD 1–20 min	POD 1- 2 h	POD 2	POD 3	Dis- charge	Follow-up
Brief Pain Inventory (± SD)	1.3 (± 2.3)	2.8 (± 2.3)	-	-	1.8 (± 1.8)	1.1 (± 1.4)	1.3 (± 2.3)	1.4 (± 2.2)
Pain Intensity Score (± SD)	1.0 (± 1.8)	2.2 (± 2.1)	-	-	1.4 (± 1.8)	0.9 (± 1.6)	0.7 (± 1.4)	0.8 (± 1.4)
Pain Interference Score (± SD)	1.3 (± 2.6)	3.7 (± 3.8)	-	-	2.8 (± 3.5)	1.7 (± 3.0)	1.9 (± 3.4)	1.5 (± 2.5)
Mean pain score at rest (± SD)	0.8 (± 1.6)	1.2 (± 1.2)	1.1 (± 1.2)	1.2 (± 1.1)	0.5 (± 0.8)	0.3 (± 0.5)	0.2 (± 0.5)	0.4 (± 0.9)
Mean of highest pain score within the last 24 h (± SD)	1.6 (± 2.5)	4.2 (± 2.6)	-	-	3.1 (± 2.4)	2.0 (± 2.5)	1.9 (± 2.1)	1.8 (± 2.1)
Mean of average pain score within the last 24 h (± SD)	1.1 (± 1.8)	2.6 (± 1.6)	-	-	1.6 (± 1.1)	1.3 (± 1.4)	0.8 (± 0.8)	0.8 (± 1.0)
NRS score at rest 0 1 2 3 4 6	18 (72%) 1 (4%) 1 (4%) 4 (16%) - 1 (4%)	10 (40%) 4 (16%) 7 (28%) 4 (16%) - -	11 (44%) 6 (24%) 2 (8%) 6 (24%) - -	9 (36%) 7 (28%) 6 (24%) 2 (8%) 1 (4%) -	10 (59%) 6 (35%) - 1 (6%) - -	9 (69%) 4 (31%) - - - -	19 (83%) 3 (13%) 1 (4%) - - -	19 (76%) 3 (23%) 1 (4%) 2 (8%) - -

*SD* Standard deviation

**Table 5:** Perioperative analgesic treatment

	Recovery room	POD 1	POD 2	POD 3	Discharge	Follow-up
Metamizol n median (mg)	1 1.000	20 1.500	13 3.000	9 3.500	14 2.000	4 as required
Paracetamolnmedian (mg)	4 1000	2 1.500	2 3.000	3 2.000	4 3.500	1 as required
Ibuprofene n median (mg)	0	0	1 1.800	1 1.800	1 1.800	0
Morphine equivalent n cumulative dose (mg) median (mg)	14 249 15,75	3 90 60	1 60 60	2 60 60	2 60 60	1 60 60

### Intervention

Acupuncture was administered on POD 1 to all participants. The specific acupuncture points selected are listed in the supplementary materials. While certain points, such as ST36, were used more frequently—appearing in nearly 50% of patients—no two interventions had identical acupuncture point selections. No significant changes in pain scores were observed at either 20 min–2 h after acupuncture. In 68% of cases, optimal point selection was limited by factors such as patient positioning, surgical dressings or wound location, or intolerance of the prone position. A second acupuncture session was scheduled for POD 3 if patients reported resting NRS pain scores above 3; however, this was not required due to generally low pain levels.

**Fig. 3:**
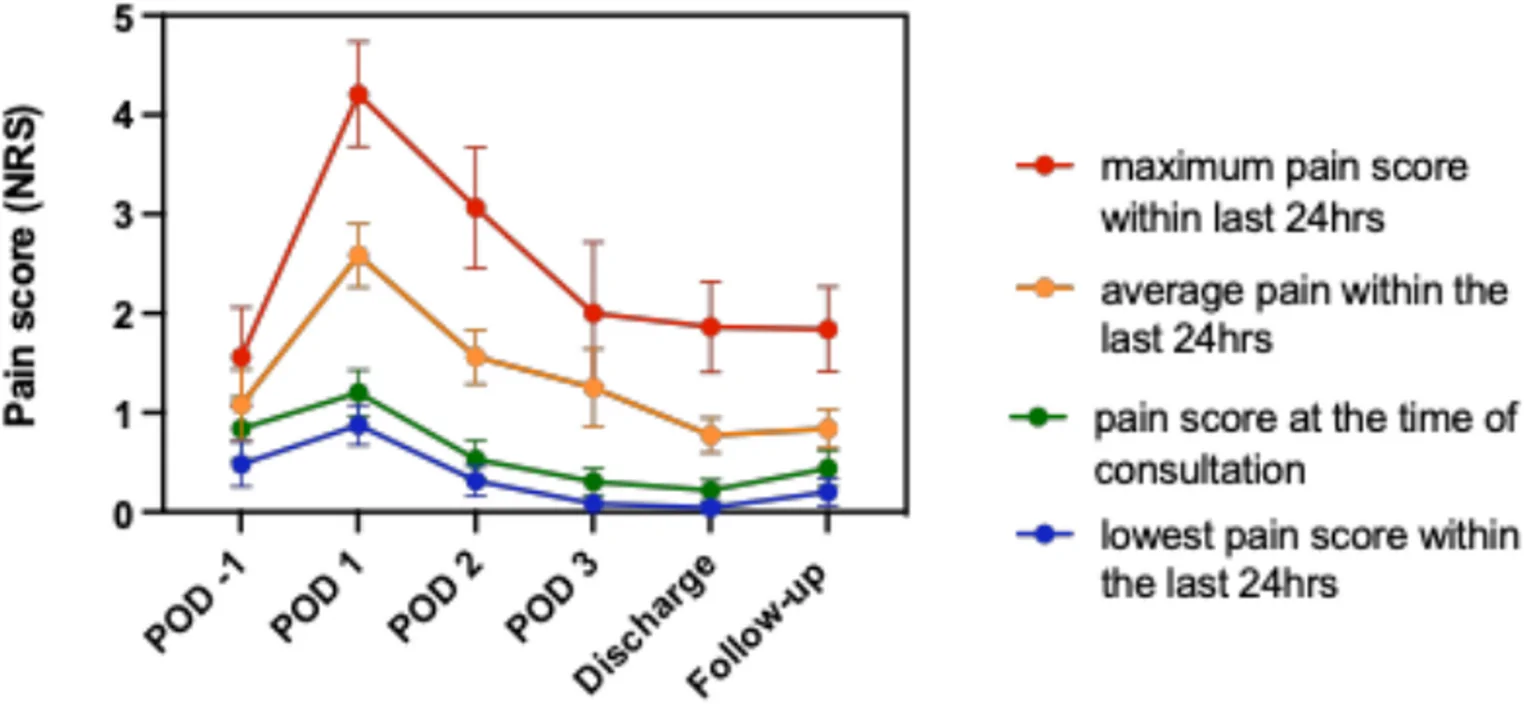
Mean pain scores during the observational period, starting preoperatively (POD − 1) and continuing until follow-up. Pain was assessed using the Numeric Rating Scale (NRS) and the Brief Pain Inventory (BPI). The green line represents the pain score recorded during daily clinical visits. The orange line shows the average pain intensity over the past 24 h, while the red line indicates the maximum pain experienced within the same period and the blue line the lowest score

Figure 3 illustrates the postoperative pain trajectory, with the highest scores recorded on POD 1, followed by a continuous decline. Similarly, average daily pain scores decreased continuously after POD 1, a trend that was also reflected in the BPI, which indicated only mild pain severity throughout the study period. Both the Pain Intensity and Pain Interference scores declined after POD 1 (Table 4). BPI scores were not significantly different from preoperative levels at any time point (*p* > 0.05) except POD 2 (*p* < 0.0001), and reached their lowest values at POD 3. Specifically, BPI did not differ between discharge and follow-up (*p* = 0.4947). The median hospital stay was four days. Only one patient required ongoing postoperative opioid therapy, having already been on opioids prior to surgery, while another patient successfully discontinued opioid use during the postoperative period.

Assessment of quality of life revealed no significant change in the Global Health Score from before to after surgery. Functional scales showed significant postoperative changes in physical, role, and emotional functioning, whereas symptom scales—including nausea, vomiting, pain, dyspnoea, insomnia, and appetite loss—did not differ significantly between the preoperative and follow-up assessments. No significant change was observed in the DASH score. In contrast, the LEFS demonstrated a significant decline during the perioperative period, indicating a worse lower limb function (Table 6).

**Table 6:** The results of the European Organization for Research and Treatment of Cancer Quality of Life Questionnaire Core 30 (EORTC QLQ-C30), Disabilities of the Arm, Shoulder, and Hand (DASH) and Lower Extremity Functional Scale (LEFS) before and after surgery are shown

EORTC QLQ-C30	Preoperative (mean ± SD)	Follow-up (mean ± SD)	P-value
Global Health Status	70.7 (± 20.3)	69.3 (± 18.7)	.99
Functional Scales:			
- Physical functioning	84.0 (± 19.1)	68.5 (± 28.8)	.0045
- Role functioning	74.0 (± 34.4)	53.3 (± 34.7)	.003
- Emotional functioning	70.7 (± 25.9)	81.0 (± 21.3)	.02
- Cognitive functioning	86.0 (± 26.2)	90.0 (± 18.0)	.27
-Social functioning	82.7 (± 25.2)	73.3 (± 33.7)	.13
Single-Item Scales:			
-Fatigue	24.4 (± 24.2)	28.0 (± 28.3)	.63
-Nausea and vomiting	0.0 (± 0.0)	0.7 (± 3.3)	1.0
-Pain	21.3 (± 27.4)	30.0 (± 28.1)	.1
-Dyspnea	12.0 (± 21.3)	5.3 (± 12.5)	.25
-Insomnia	26.7 (± 27.2)	17.3 (± 25.7)	.12
-Appetite loss	6.7 (± 13.6)	5.3 (± 12.5)	1.0
-Constipation	4.0 (± 11.1)	12.0 (± 28.7)	.25
-Diarrhea	1.3 (± 6.7)	2.7 (± 9.2)	1.0
-Financial difficulties	8.0 (± 24.1)	8.0 (± 17.4)	1.0
DASH main module	20.0 (± 18.6)	12.5 (± 11.4)	-
LEFS	63.9 (± 17.6)	44.7 (± 20.2)	.0001

*SD* Standard deviation

### Postoperative complications

Of the 25 patients, eleven developed postoperative wound healing complications during the study period. In 6 cases, prolonged secretion via the drainage without the need for further treatment, occurred. Two required minor re-interventions, such as seroma aspiration. Two patients required re-hospitalization for treating lymphocele (*n* = 1) or wound complication (*n* = 1).

## Discussion

To our knowledge, this was the first study to investigate postoperative acupuncture treatment in patients with STT, and to prospectively document both pain levels and analgesic consumption in the immediate postoperative period following surgery for extremity tumors.

Overall, our findings indicatet that patients experienced consistently low pain levels throughout the postoperative period, which was reflected by both low NRS scores and low BPI values. Consequently, we discontinued the feasibility study after enrolling 25 patients instead of the initially planned 100. Acupuncture was administered to all patients on POD 1 according to protocol. A second session was scheduled for POD 3 if pain levels exceeded an NRS ≥ 3; however, no patient met this criterion, rendering further intervention unnecessary. Notably, the highest average and maximum 24-hour pain scores occurred on POD 1. Overall, these findings also indicate that early discharge, with a median hospital stay of four days, was feasible and safe in this cohort.

Regarding the feasibility of a non-pharmacological pain treatment approach, optimal acupuncture point application was achievable in 32% of cases; mainly limitations were wound dressings or patients’ inability to assume the prone position. This raises the question of whether a wound management strategy could be developed that meets hygiene standards while allowing adequate access to acupuncture points. It also warrants consideration whether acupuncture applied outside predefined locations could achieve comparable effectiveness. Furthermore, the feasibility of preoperative marking of acupuncture points—taking into account the planned surgical incision—should be explored.

Postoperative quality of life, assessed with the EORTC QLQ-C30, showed stable global health scores from pre- to post-surgery, although functional scales declined for some items postoperatively. Our patients demonstrated slightly better functional outcomes (mean score 69) compared to the PROSa study cohort for lower extremity STS patients (mean score 63), while the PROSa cohort reported a marginally higher global health score (73 vs. 69.3 in our study). Symptom burden in our cohort was lower across most domains, including nausea/vomiting (0.7 vs. 5.7), pain (30.0 vs. 35.3), dyspnoea (5.3 vs. 22.4), insomnia (17.3 vs. 36.9), appetite loss (5.3 vs. 13.6), diarrhoea (2.7 vs. 8.2), and financial difficulties (8.0 vs. 21.7). Constipation scores were comparable (12 in both cohorts) [34]. Quality of life in the immediate postoperative period was assessed exploratorily, revealing a general symptom burden across multiple domains. These results underscore the importance of optimised pain management. Kruiswijk et al. evaluated long-term postoperative outcomes after high-grade extremity STS surgery over a 12-month period and reported good functional recovery of the operated limb, while mental and emotional functioning showed only minimal improvement during this interval [35]. In contrast, patients in our cohort demonstrated substantially greater emotional functioning during follow-up.

Patients with chronic pre-existing pain may particularly benefit from adjunctive therapies such as acupuncture, especially when these interventions also address co-occurring symptoms, including psychological distress [36, 37]. Notably, the majority of patients in our cohort (64%) reported prior experience with complementary medicine, underscoring the relevance of further research into such treatment approaches.

This study has some limitations, including the small sample size and the absence of a control group. Therefore, a causal attribution to acupuncture treatment is not possible. Nonetheless, these findings are encouraging and support current clinical practice, as they provide the first insights into postoperative treatment of STTs of the extremities. For future studies, particular attention should be given to patients with preoperative pain, as they also reported higher postoperative pain levels. This subgroup as well as those patients with high pain levels may be the most suitable for evaluating the effectiveness of postoperative acupuncture.

## Conclusion

This feasibility study highlights the challenges of implementing postoperative acupuncture after STT resection.

Pain levels remained low throughout the perioperative course, with minimal opioid requirements. Although the study was not designed to assess efficacy, the observed pain trajectory—with a peak on POD 1 followed by a significant reduction—indicates that postoperative pain was generally well controlled. We did generate data on how much pain patients after STT resection of the extremities experienced and how much analgesics were required within multimodal approach.

Notably, no acupuncture-related complications were observed. Future studies should evaluate both, postoperative and preoperative acupuncture, particularly in patient subgroups likely to experience higher pain levels. In the present study, the need for adjunctive pain management with acupuncture is questionable given the generally low postoperative pain scores. However, patients with chronic pain may represent a more suitable target population for future research.

## Data Availability

No datasets were generated or analysed during the current study.
